# Management of PTLD After Hematopoietic Stem Cell Transplantation: Immunological Perspectives

**DOI:** 10.3389/fimmu.2020.567020

**Published:** 2020-09-16

**Authors:** Francesca Compagno, Sabrina Basso, Arianna Panigari, Jessica Bagnarino, Luca Stoppini, Alessandra Maiello, Tommaso Mina, Paola Zelini, Cesare Perotti, Fausto Baldanti, Marco Zecca, Patrizia Comoli

**Affiliations:** ^1^Pediatric Hematology/Oncology, Fondazione Istituto di Ricovero e Cura a Carattere Scientifico Policlinico San Matteo, Pavia, Italy; ^2^Cell Factory, Fondazione Istituto di Ricovero e Cura a Carattere Scientifico Policlinico San Matteo, Pavia, Italy; ^3^Immunohematology and Transfusion Service, Fondazione Istituto di Ricovero e Cura a Carattere Scientifico Policlinico San Matteo, Pavia, Italy; ^4^Virology Service, Fondazione Istituto di Ricovero e Cura a Carattere Scientifico Policlinico San Matteo, University of Pavia, Pavia, Italy

**Keywords:** epstein-barr virus, T cell immunity, virological monitoring, prophylaxis, preemptive treatment

## Abstract

Post-transplant lymphoproliferative disorders (PTLDs) are life-threatening complications of iatrogenic immune impairment after allogeneic hematopoietic stem cell transplantation (HSCT). In the pediatric setting, the majority of PTLDs are related to the Epstein–Barr virus (EBV) infection, and present as B-cell lymphoproliferations. Although considered rare events, PTLDs have been increasingly observed with the widening application of HSCT from alternative sources, including cord blood and HLA-haploidentical stem cell grafts, and the use of novel agents for the prevention and treatment of rejection and graft-vs.-host disease. The higher frequency initially paralleled a poor outcome, due to limited therapeutic options, and scarcity of controlled trials in a rare disease context. In the last 2 decades, insight into the relationship between EBV and the immune system, and advances in early diagnosis, monitoring and treatment have changed the approach to the management of PTLDs after HSCT, and significantly ameliorated the prognosis. In this review, we summarize literature on the impact of combined viro-immunologic assessment on PTLD management, describe the various strategies for PTLD prevention and preemptive/curative treatment, and discuss the potential of novel immune-based therapies in the containment of this malignant complication.

## Introduction

Post-transplant lymphoproliferative disorders (PTLD) are heterogeneous lymphoproliferative diseases that stem from the unchecked proliferation of neoplastic lymphoid or plasmacytic cells in the setting of immunosuppression after transplantation (1–3).

PTLD in the hematopoietic stem cell transplantation (HSCT) setting are almost exclusively related to Epstein-Barr virus (EBV) infection; they usually develop between 3 and 6 months post-transplant, when virus-specific T cell immunity has not yet reconstituted, and are generally of donor origin. Although recipient-derived PTLDs have been described, they occur mainly in patients with poor graft reconstitution.

This review outlines our current understanding of the interplay between the virus and the immune system in the pathogenesis of these disorders after HSCT, and how our knowledge has improved current approaches to the management of PTLD in this clinical setting.

## Interactions Between EBV And the Host

EBV is a human γ-herpesvirus that infects more than 90% of the individuals worldwide (4–6). The virus enters the organism through the oropharyngeal route, and, in healthy subjects, causes a self-limiting primary infection. In normal, seropositive individuals, virus neutralizing antibodies control the spread of infectious virus particles and EBV-specific, HLA class I restricted, CD8+ cytotoxic T lymphocytes (CTL) specific for the early lytic cycle proteins kill cells entering the lytic cycle before they are able to release infectious virus particles (7).

The virus is B lymphotropic, and persists in resting memory B cells for the lifetime of the host in a non-pathogenic state that is invisible to the immune response (8). Initially, EBV infects naïve B cells in tonsillar lymphoepithelium, driving their activation through the expression of nine latent proteins (EBV nuclear antigens, EBNAs 1, 2, 3a, 3b, 3c, and LP and membrane antigens LMP 1, 2a and 2b), two small non-translated RNAs and about 40 microRNAs that constitute the EBV growth program (9). CTL directed to EBV latent cycle antigens prevent the outgrowth of cells latently infected with the virus (7).

Thence, the virus biology parallels that of normal mature B lymphocytes. EBV-infected naïve B-cells migrate to germinal centers in lymph nodes, lymphoid tissue present in mucosa, or the spleen. In germinal centers, normal B-cells undergo activation-induced somatic hypermutation and class switch recombination of the antigen-binding variable region of immunoglobulin genes. Within the germinal center, EBV-positive B cells shift to a more restricted virus transcription program, the default program (EBNA1, LMP1, and LMP2a expression), that helps rescue them into the memory compartment where the virus persists (6). Expression of viral proteins provides EBV-infected naïve B-cells with a selective advantage in the germinal center, and stimulates maturation into memory B-cells, which are the presumed reservoir of EBV (10).

Memory cells latently infected with EBV in the peripheral blood are in the latency program, and do not express any of the known latent proteins, unless they undergo division, in which case they express EBNA1, essential for the maintenance of the viral episome in dividing cells (8, 9). The frequency of infected memory B cells in a healthy carrier is stable over time, although it varies among different individuals, and has been calculated to be around 0.5 × 10^6^, with only 1% residing in the peripheral blood (10). The virus is no longer pathogenic to the host, as the genes that drive cellular proliferation and may lead to neoplastic disease are no longer expressed. Likewise, the virus is safe from immune surveillance, as immunogenic viral protein expression, which serves as a target for the immune system is absent.

## Interplay Between EBV and the Immune System: Pathogenesis of PTLD After HSCT

EBV is considered an oncogenic virus, because of its association with tumors. EBV has latent proteins that can drive cellular proliferation, at least in B lymphocytes, such as LMP1 and LMP2, and these likely play a causative role in tumor development through inappropriate or deregulated gene expression (4, 8). HSCT recipients have impaired T-cell mediated immunity due to the pre-transplant conditioning regimen, immunosuppressive agents for prophylaxis of graft-vs.-host disease (GVHD) and GVHD itself (11–16). The reduced numbers of EBV-specific CTLs facilitate uninhibited growth of EBV-infected cells (17, 18). However, only a small number of EBV-positive patients develop PTLD after HSCT or other conditions of immunosuppression (11), and advanced PTLD is an oligoclonal rather than polyclonal disease, suggesting that other rare events contribute to the pathogenesis of the disorder. Thus, in order to have PTLD development, the growth program must be erroneously expressed in a B cell that cannot exit the cell cycle, and immunosuppression must prevent the elimination of these rare cells. At this stage, the disease may still be controlled by intervening on the immune status. Indeed, PTLD patients in early stages of disease may regress in response to the reduction of immunosuppression (19, 20) or after donor lymphocyte (DLI) (21) or EBV-specific CTL infusion (18, 22), strongly pointing to the essential role played by the underlying state of immunosuppression. However, in the absence of T cell immunity, such as it is often observed after T-cell depleted HSCT, proliferating cells acquire additional genetic or epigenetic damage, and these new cell clones may become unresponsive to immune surveillance (23).

A similar pathogenetic mechanism may be hypothesized in the rare cases of EBV-positive PTLD of T-cell origin. It has been postulated that some T cells may express CD21, the EBV receptor on B cells, and thus may allow viral entry (24).

## Risk Factors FOR EBV-PTLD After HSCT

Development of PTLD after HSCT is mainly associated with T-cell depletion of the graft before transplantation and the type/duration of immunosuppression employed to prevent and treat graft-vs.-host disease, and the degree of mismatch between recipient and donor (1, 2, 25–28). Consequently, PTLD is more often observed in T-cell depleted HSCT from haploidentical donors.

Among *ex-vivo* approaches, elective T cell depletion methods are associated with a greater increase in PTLD risk (26), as donor EBV-targeted cytotoxic T cells are removed from the inoculum, thus compromising specific immune surveillance. However, the use of lymphocyte depletion strategies that target both T and B cells, such as *in vitro* alemtuzumab (26) or combined depletion of αβ T-cell and CD19 B-cells (29), have a lower risk of PTLD, by delaying potential EBV-infected B cell proliferation until recovery of functional T cell immunity. This observation supports the concept that an imbalance between EBV-infected B cells and EBV-specific T cells favors neoplastic outgrowth of EBV-positive B cells. Likewise, *in vivo* depletion of T-cells using antithymocyte globulin (ATG) is associated with a higher risk of developing PTLD than the use of broad lymphocyte-targeting alemtuzumab monoclonal antibody (Mab) (26, 30, 31). Rabbit ATG was suggested to be more likely to cause profound lymphodepletion than horse ATG (32). However, a recent study in the setting of pediatric and adult haploidentical HSCT show comparable rates of EBV DNAemia and PTLD (33). The effect of ATG seems dose-dependent, as high-dose ATG had a 2.3-fold higher risk of PTLD than low-dose (30, 34).

The degree of HLA matching between donor and recipient correlates with the development of PTLD. HSCT from a HLA-mismatched donor has been observed to be associated with a higher risk of PTLD than the use of a HLA-identical donor (26, 27). Although a certain degree of mismatch between recipient and donor may impair EBV antigen recognition by HLA-restricted donor T cells, the risk associated with HLA mismatch is mainly due to the *in vitro* and/or *in vivo* T-cell depletion strategies employed in mismatched transplants to facilitate engraftment and prevent GVHD: the combination of different depletion approaches results in additive risk (26).

The incidence of PTLD with regard to the different types of donors ranges from 1% in HSCT from matched related donors to 4% for matched unrelated and 11% for mismatched unrelated donors (20). Among different stem cell sources, cord blood was associated with a greater risk of PTLD (30), due to low numbers and naiveté of infused T cells that likely delay early immune reconstitution, although there is no evidence of delayed virus-specific immune recovery in pediatric CBT recipients beyond the first 100 days post-transplant (35). Moreover, there is *in vitro* evidence that CB lymphocytes may mediate a sizeable immune response directed against autologous EBV-infected cells, exerted by both NK cells and CD4+ T lymphocytes (36). The development of graft engineering strategies and pharmacologic GVHD prevention protocols, together with optimal conditioning regimens, have significantly ameliorated the outcomes of haploidentical HSCT, and this progress has led to a widespread use of the procedure (18, 29, 37–46). Interestingly, despite the high degree of mismatch and the procedures employed to facilitate engraftment and prevent GVHD, the incidence of PTLD with the newest platforms for haplo-HSCT, either T-cell and B-cell depleted (29, 43) or T-cell repleted with post-transplant cyclophosphamide (PTCy) (44–46), are unexpectedly low. In the case of PTCy, the incidence is <3% (47), possibly due to the destruction of EBV-infected B cells, together with an immune reconstitution that is hypothesized to be faster than that observed after the use of ATG (48).

Among other risk factors relevant for pediatric HSCT recipients, a higher incidence of PTLD has been observed in recipients of allogeneic HSCT conditioned with a reduced-intensity regimen (49), and the development of acute or chronic GVHD (20, 26, 27, 30), due to a delay in the reconstitution of functional specific immunity. Finally, EBV serology mismatch, in particular EBV-seronegative patients receiving grafts from seropositive donors, are also at increased risk for PTLD development (25, 27).

Some studies have suggested that significant factors could be combined within a prognostic model. Three single-center risk factor scoring systems have been published, but their use in common clinical practice is limited and needs to be validated (25, 27, 30).

## Epidemiology, Clinical Presentation and Diagnosis

EBV-PTLD is a severe complication that occurs in 1–3.5% of HSCT recipients (20, 50), although incidence rates may exceed 10% in patients with established risk factors (2, 20, 27, 28, 50). An expansion in the indications for HSCT from alternative donors, including haploidentical family members, and the use of novel T-cell depletion strategies, together with improved diagnostic protocols, have led to the observation of an increased incidence of PTLD in the last 2 decades (20). However, greater awareness of the disorder has fueled studies that have addressed PTLD preemptive/preventive strategies, and facilitated patient management.

Patients with PTLD after HSCT generally present with fever, lymphadenopathy, tonsil enlargement or discrete organ lesions, although the disease may manifest as a systemic process that mimics fulminant sepsis syndrome (2, 28). Primary central nervous system (CNS) localization of PTLD is rare, and generally burdened with a dismal prognosis (20), partly due to the challenges associated with limited drug penetration across the blood-brain barrier. In order to overcome this peculiar feature, intrathecal drug delivery has been proposed (51).

The diagnosis of EBV-PTLD is based on symptoms and/or signs consistent with PTLD, together with the quantitative determination of EBV-DNAemia or detection of EBV in a specimen from the involved tissue (1, 2), and imaging studies, such as computed tomography (CT) or positron emission tomography CT (PET-CT). Definitive diagnosis of EBV-PTLD requires biopsy of sites suspected for EBV disease and histological examination. EBV detection requires identification of viral antigens or *in situ* hybridization for the EBER transcripts. The histological WHO 2016 classification includes six morphological types of PTLD: plasmacytic hyperplasia, infectious mononucleosis-like, florid follicular hyperplasia, polymorphic, monomorphic (B-cell or T-/NK-cell types), and classical Hodgkin lymphoma PTLD (52).

EBV-PTLD may be diagnosed at the probable or proven level (53). Probable EBV disease is defined as the presence of symptoms and/or signs of lymphoproliferative disease in the absence of tissue biopsy, but without other documented causes, together with significant EBV DNAemia, measured in any blood specimen. Diagnosis of proven EBV-PTLD requires detection of EBV nucleic acids or EBV-encoded proteins in a tissue sample.

## Early Identification of Patients at Risk of PTLD

The development of EBV-PTLD after HSCT represents a life threatening event; mortality is still relevant, at 30 and 40% of diagnosed cases (20, 54). The onset of PTLD is preceded by a pre-clinical phase denoted by increased EBV DNA levels in the peripheral blood. Indeed, it has been demonstrated that, irrespective of baseline characteristics, the post-transplant monitoring of peripheral EBV viral load after HSCT is effective in predicting risk of EBV-PTLD (18, 55–61).

Thus, according to international guidelines, prospective monitoring of EBV DNA should be started within the first month after HSCT, and continued on a weekly basis at least until the fourth post-transplant month (1). The frequency and duration of EBV DNAemia screening should be based on the risk profile of the transplanted patients (62).

EBV DNA analysis is an indispensable tool for early diagnosis and the application of preemptive strategies to avoid progression of early-stage PTLD to oligoclonal/monoclonal disease (18, 63). However, even with the available data there is not a defined EBV-DNA threshold for prompt initiation of preemptive therapy (1), as EBV PCR assays are not standardized (63), and evidence has been obtained in cohorts with heterogeneous clinical characteristics using different peripheral blood specimens. Thresholds for assays using mononuclear cells, plasma, or whole blood in the reported studies range from 1,000 to 40.000 copies/ml according to the source, and data on the best specimen source are inconclusive (1, 59–61, 64, 65). Moreover, probable/proven PTLD has been described in a proportion of patients with EBV DNA levels below commonly adopted intervention thresholds (66, 67). Thus, it seems rational to adopt validated center-specific cut-off values, tailored on the specific cohort characteristics, and employ the rate of EBV DNA level increase, that is an indicator of EBV-infected B cells, as a predictor of when to start preemptive interventions. Regarding peripheral blood specimen choice, a recent study including 121 pediatric and adult HSCT recipients evaluated the kinetics of EBV DNA, assessed with a molecular method approved by regulatory agencies, in paired whole blood and plasma samples during episodes of post-transplant EBV infection, and found that plasma had a low sensitivity for identifying PTLD, thus suggesting a preferential use for whole blood in the post-transplant management of infection (64). Some studies indicated that plasma measurement may be useful in the follow-up after treatment, but these studies included high numbers of solid organ transplant recipients, and data are yet not conclusive (60).

EBV DNA analysis is not a precise predictor of PTLD development, and tailoring screening on the basis of a whole cohort is not always practical, feasible, or successful. As the other central factor determining progression to PTLD is the lack of a protective immune response, it seems reasonable to associate DNAemia screening with analysis of immune reconstitution. This approach has been used successfully for other viral infections in HSCT or solid organ transplant patients (13, 68–75), and has been proposed by some groups also in the setting of EBV infection and PTLD after HSCT (18, 76–82). Although studies are largely descriptive and based on the use of different technologies, the results suggest that numbers and function of virus-specific T cells inversely correlate with viral DNA levels and risk of disease, whereby strong cellular immune responses are associated with containment of viral replication or EBV-infected B cell outgrowth. The key obstacles to the introduction of EBV-specific T cell quantification into clinical practice is the definition of reliable cutoffs for clinical decision making for the different assays, and the absence of controlled interventional clinical trials.

## Prevention of PTLD After HSCT

There are two possible approaches for prevention of EBV-PTLD after HSCT: prophylaxis and pre-emptive therapy (53, 83, 84). Prophylaxis of EBV disease includes any intervention applied to an asymptomatic patient to prevent EBV DNAemia. Pre-emptive therapy includes any intervention given to a patient with EBV DNAemia to prevent EBV disease.

### Prophylaxis

In the setting of HSCT, there are two strategies to prevent EBV DNAemia. The first is based on interventions on the graft or the patient prior to HSCT, in order to decrease the risk of EBV-infected B cell outgrowth. As we have already seen, in the case of T-cell depleted HSCT, the use of *in vitro* or *in vivo* methods that deplete B cells as well as T cells reduce the risk of PTLD by temporarily removing the EBV reservoir and potential EBV-transformed B lymphoblasts, at least until functional immune reconstitution is achieved (28, 29, 43, 44, 47). If no T-cell depletion is employed, but the risk of PTLD is high due to the use of ATG and/or the presence of HLA mismatches between donor and recipient, peri-transplant B-cell depletion by rituximab may be considered (85). The efficacy of peritransplant rituximab was suggested by observations in adult patients receiving anti-CD20 monoclonal antibody close to HSCT, as part of their treatment for B cell malignancies (85), and was tested in a study from the European Group for Blood and Marrow Transplantation (EBMT) as part of the conditioning regimen for pediatric and adult patients with severe aplastic anemia (86). Based on these studies, peritransplant rituximab has been employed in pediatric recipients of αβ T-cell/B-cell depleted haploidentical HSCT, and the combination of *in vitro* and *in vivo* B cell depletion succeeded in counteracting the risk of PTLD given by T-cell depletion, ATG and HLA mismatch (29, 87). Relevantly, rituximab role in controlling acute and chronic GVHD may also favorably impact PTLD development (29, 85–87).

Regarding the use of ATG to prevent rejection and GVHD, given that the increased risk of PTLD is dose-dependent, in pediatric allogeneic HSCT a lower therapeutic dose may be administered. Indeed, a recent multicenter randomized trial has shown that 15 vs. 30 mg/kg rabbit ATG was equally effective in preventing acute and chronic GVHD, but was associated with lower relapse and non-relapse mortality in pediatric patients receiving UD HSCT for hematologic malignancies (88). An alternative GVHD prophylaxis, that may have a direct impact on PTLD development due to its anti-tumor activity, is the use of mTOR inhibitors (89, 90).

Post-transplant prophylactic administration of agents, such as antiviral drugs, rituximab and EBV-CTLs has been proposed. Treatment of latent EBV with antivirals has been unsuccessful, as latently infected B cells do not express the EBV thymidine kinase enzyme (1, 84).

In a large retrospective study, prophylactic post-transplant rituximab significantly reduced the risk of EBV DNAemia (91); however, no statistically significant impact on PTLD incidence, treatment-related mortality, and overall survival in comparison to a pre-emptive approach was shown. Post-transplant rituximab is associated with cytopenias (92) and delayed B cell recovery with an increased risk of infections (93), that seem less evident with peritransplant use. Thus, prophylactic rituximab post-HSCT ought to be employed with caution (1). The use of prophylactic EBV-CTLs, pioneered by Rooney et al. in high-risk, pediatric unrelated-donor HSCT recipients, has been highly successful, and devoid of side effects (22, 94) (Table 1). None of the 101 patients who received CTLs as prophylaxis developed PTLD compared with 11.5% of controls. As the donors were EBV-seropositive, even in the absence of circulating EBV one may hypothesize that the efficacy of this treatment was due to stimulation by EBV present in patient tissues or donor B cells, or just cross-stimulation of low-affinity T cells present in the infused product by other antigens. Current use of EBV-CTLs is, however, limited to a few selected centers.

**Table 1:** Results of published trials using EBV-specific T cells to prevent or treat EBV infection and PTLD.

**References**	**Pt n**.	**EBV stimulation (other targeted viruses)**	**Clinical design**	**Clinical and virologic effects on EBV and PTLD**	**GVHD**
**HSCT donor-derived, single-VST**					
Rooney et al. (22) and Heslop et al. (94)	113	EBV-LCL	Prophylaxis	11/13 pts achieved CR, none PTLD	8/51 pts aGvHD; 13/108 cGvHD (11 limited, 2 extensive)
Doubrovina et al. (95)	14	EBV-LCL	PTLD Treatment	10 pts achieved CR, 4 pts progressive disease	None
Gustafsson et al. (96)	6	EBV-LCL	Pre-emptive	5 pts had EBV-DNA decreased, 1 pts died of PTLD	None
Lucas et al. (56)	1	EBV-LCL	PTLD treatment	CR	Limited skin aGvHD
Imashuku et al. (97)	1	EBV-LCL	PTLD treatment	No response	None
Comoli et al. (18)	4	EBV-LCL	Preemptive or PTLD treatment	3 pts achieved CR, 1 pt had decreased EBV-DNA level without PLTD	None
Moosmann et al. (98)	6	Peptide mix from lytic and latent EBV antigens	PTLD treatment	3 pts had CR, 3 pts had no response	None
Icheva et al. (99)	10	Recombinant EBNA1 protein or EBNA1 peptides and direct selection	Pre-emptive or PTLD treatment	7/10 pts achieved CR	1 grade II aGVHD
Jiang et al. (100)	15	DCs pulsed with EBV-LCL lysate	PTLD treatment (+rituximab and/or CHOP)	7/8 pts achieved CR	5 pts (33%) aGVHD (1 gr. I, 3 gr. II, 1 gr. III) 2 (13%) limited cGVHD
Velvet et al. (101)	2	unknown	CNS-PTLD treatment	1 pt achieved remission	None
**HSCT donor-derived, multi-VST**					
Leen et al. (102) and Melenhorst et al. (103)	26	EBV LCLs transduced with Ad5f35-pp65 (ADV, CMV)	Prophylaxis/preemptive	6/6 pts with EBV cleared infection;	2 grade I aGVHD
Leen et al. (104)	14	EBV LCLs transduced with Ad5f35 vector (ADV)	Prophylaxis	11 pts treated as prophylaxis remain negative	3 grade I aGVHD
Dong et al. (105)	3	DCs pulsed with EBV IE1 and LMP2 peptides (CMV)	Prophylaxis/preemptive	1 pt cleared viremia; 1 pt treated as prophylaxis remains negative	1 grade I aGVHD
Gerdemann et al. (106)	10	DCs nucleofected with plasmids encoding for EBV LMP2 and BZLF1 (ADV, CMV)	Preemptive/PTLD treatment	3/4 pt: complete virologic responses	1 skin rash due to GVHD or BKPyV infection
Papadopoulou et al. (107)	11	Peptides pool from immunodominant antigens (ADV, CMV, PyVBK, HHV6)	Prophylaxis/preemptive	3 pts treated as prophylaxis remain negative; 4/4 pts cleared EBV viremia	1 grade I aGVHD
Ma et al. (108)	10	Ad5f35-EBNA1/LMP (ADV, CMV, VZV)	Prophylaxis	no EBV reactivation	1 grade II aGVHD 1 grade III aGVHD
**Third-party donor-derived single-VST**					
Haque et al. (109)	33	EBV-LCL	PTLD treatment	14 pts attained EBV CR, 3 pts had PR, 16 pts no response at 6 m	None
Barker et al. (110)	5	EBV-LCL	PTLD treatment	4 pts attained EBV CR, 1 pts progressive disease	None
Uhlin et al. (111)	1	Peptide-HLA multimer selection	Preventive and PTLD treatment	CR after 9 m, recurrence then response to 2nd infusion	None
Prockop et al. (112)	33	EBV-LCL	PTLD treatment	CR or PR was achieved in 68% of HSCT recipients. For patients who achieved CR/PR or SD after cycle 1, 1y OS was 88.9%	1 grade I skin aGvHD
**Third-party donor-derived multi-VST**					
Leen et al. (113)	50	LCLs transduced with Ad5f35-pp65 (ADV, CMV)	PTLD treatment	6/9 pts with EBV attained CR or PR;	6 grade I aGVHD 2 grade II aGVHD
Tzannou et al. (114)	38	EBV LMP2 + EBNA1 + BZLF1 peptide pools (ADV, CMV, PyVBK, HHV6)	Preemptive/PTLD treatment	3/3 pts with EBV attained CR;	2 grade I aGVHD de novo; 4 grade I-III recurrent aGVHD

### Preemptive Therapy

The mainstay of pre-emptive therapy for EBV PTLD after HSCT is anti-CD20 antibody rituximab, given at increase in EBV-DNA load especially in patients lacking T-cell reconstitution (18, 63, 86, 115), due to its acceptable toxicity and widespread availability (1, 84). A retrospective study reviewed the results of more than 300 patients described in reported case series, and found that successful prevention of PTLD was observed in almost 90% of treated patients (84). Pre-emptive rituximab is employed at the dose of 375 mg/m^2^, once weekly until EBV DNAemia is found negative. Dose number should be assessed on the basis of EBV DNAemia monitoring and on the patient's specific immune recovery, but 1–4 doses are generally sufficient. However, it has been shown that, in certain circumstances, clearance of EBV DNA from peripheral blood may not reflect a long-lasting response (67). Limitations of this approach are selection for CD20-negative clones (18), and the fact that rituximab acts on the EBV reservoir, rather than on restoration of the cellular immune response to EBV, which is central to the long-term control of EBV mediated B-cell proliferation (18, 72).

Among the strategies that boost specific immune reconstitution and immune surveillance, reduction of maintenance immunosuppression (IS), whenever feasible in the absence of GVHD, should be employed in association with anti-CD20 Mab therapy (1). Data on IS reduction employed alone are too limited to derive any useful indications (19). The use of donor EBV-specific T cells in unrelated donor (UD) or haplo-HSCT in a pre-emptive approach has been very successful (18, 96, 116–119), with long-lasting EBV viral load clearance in more than 90% of patients, and responses observed also in patients with increased viral load after rituximab treatment (18) (Table 1). In HSCT recipients, EBV-CTL therapy enhances virus-specific immune responses, and allows establishment of a memory T cell response, observed for as long as 9 years after T cell administration (117). No major toxicity was observed (118), and the reported rate of new-onset GVHD was around 1% (119). When the donor is not available, or is EBV-seronegative, or to increase access to T cell treatment, the use of third-party CTLs has been advocated (109). The first reported study used banked EBV-specific CTLs to treat PTLD after solid organ or HSCT, matching by low resolution HLA typing and screening for absence of alloreactivity, obtaining 50% responses in established PTLD and no GVHD development (109). Since then, a number of studies have further explored this option and refined matching criteria by evaluating activity against viral epitopes through the shared HLA allele (110–114, 119) (Table 1). A recent study treated 33 HSCT recipients with third-party CTLs, obtaining a 68% remission rate, with a 89% overall survival. Interestingly, patients in progression after the first cycle benefited from a change in CTL donor (9% survival after repeated cycles with same donor CTLs vs. 60% survival after donor switch) (112).

## Conclusions

Prognosis of EBV PTLD after HSCT is still suboptimal. Because of the relatively low incidence of this complication, and its particular situation related to the post-HSCT period, there is limited evidence on the best treatment strategy for established disease failing first line treatment.

Thus, therapeutic strategies with high efficacy and minimal toxic effects for HSCT patients at high risk of PTLD are a clinical need. Knowledge on the interplay between the virus and the host immune system (120) has allowed the design of tailored management approaches, based on longitudinal combined virological and immunological testing, and the development of novel cellular therapeutic agents burdened with little toxicity, and therefore suitable for employment in pre-emptive therapeutic strategies. Limitations to the pre-emptive approach are related to the difficulty in establishing viral load cut-off values for the start and discontinuation of therapeutic interventions, and standardized and cellular immunity assays with validated thresholds, together with limited availability of cellular therapies. These hurdles may be overcome by a general effort in standardization, which has already begun, and by local management. So far, the use of EBV-specific T cells has been limited to the few academic centers with infrastructure resources to produce advanced cellular therapies. Recently, excellent cell therapy clinical results, together with the development of new methodologies to obtain rapid manufacture of third-party T cells, have fuelled considerable interest from the Pharmaceutical industry to bring to the market third-party cellular therapies including EBV CTLs. Further efforts are required to design the most appropriate clinical trials to rapidly identify efficient combinatorial approaches, and to invent new and sustainable reimbursement modalities for novel therapies.

## Author Contributions

FC, SB, AP, JB, LS, AM, TM, PZ, CP, FB, MZ, and PC all participated in writing the manuscript. FC, SB, MZ, and PC co-edited the final version of the manuscript. All authors have read and approved the final manuscript.

## Conflict of Interest

The authors declare that the research was conducted in the absence of any commercial or financial relationships that could be construed as a potential conflict of interest.

## Abbreviations

PTLD: Post-transplant lymphoproliferative disorders
HSCT: hematopoietic stem cell transplantation
EBV: Epstein-Barr virus
CTL: Cytotoxic T lymphocytes
a/cGVHD: acute/chronic graft-vs.-host disease
ATG: antithymocyte globulin
Mab: monoclonal antibody
CBT: cord blood transplantation
PTCy: post-transplant cyclophosphamide
PCR: polymerase chain reaction
IS: immunosuppression
UD: unrelated donor.
